# Emotion Processing in Children with Conduct Problems and Callous-Unemotional Traits: An Investigation of Speed, Accuracy, and Attention

**DOI:** 10.1007/s10578-020-00976-9

**Published:** 2020-03-13

**Authors:** Daniela Hartmann, Christina Schwenck

**Affiliations:** Department of Special Needs Educational and Clinical Child and Adolescent Psychology, Justus-Liebig-University of Giessen, Otto-Behaghel-Straße 10 C, 35394 Giessen, Germany

**Keywords:** Conduct disorder, Oppositional defiant disorder, Callous-unemotional traits, Eye gaze, Emotion processing

## Abstract

This study aimed to assess whether callous-unemotional traits (CU) are associated with deficits in emotion recognition independent of externalizing behavior and whether such deficits can be explained by aberrant attention. As previous studies have produced inconsistent results, the current study included two different emotion recognition paradigms and assessed the potential influence of factors such as processing speed and attention. The study included N = 94 children (eight to 14 years) with an oversampling of children with conduct problems (CP) and varying levels of CU-traits. Independent of externalizing behavior, CU-traits were associated with slower recognition of angry, sad and fearful facial expressions but not with higher error rates. There was no evidence that the association between CU-traits and emotion processing could be explained by misguided attention. Our results implicate that in children with high levels of CU-traits emotion recognition deficits depend on deficits in processing speed.

**Electronic Supplementary Material:**

The online version of this article (10.1007/s10578-020-00976-9) contains supplementary material, which is available to authorized users.

## Introduction

Aside from body posture and hand gestures, facial expressions are probably one of the most important aspects of non-verbal communication. Properly decoding facial expressions is crucial in everyday situations and especially in potential conflict situations, as they can inform us about the emotions and feelings of other individuals. Deficits in recognizing negative emotions have been repeatedly linked to various problem behaviors of children with conduct disorder (CD) and oppositional defiant disorder (ODD) [1]. According to the DSM-5 [2], children with such conduct problems (CP) show externalizing problem behavior, including aggression, deceitfulness, violations of rules and social norms, argumentative/defiant behavior, and vindictiveness. It is estimated that almost half of the individuals with CP exhibit high levels of callous-unemotional traits (CU-traits) [3]. CU-traits are characterized by a lack of empathy, shallow affect, a lack of remorse or guilt, and indifference towards one’s performance. High levels of CU-traits also occur in about two to five percent of otherwise healthy individuals [4]. High levels of CU-traits can also be observed in adults with psychopathy, as they build the affective dimension of the construct of psychopathy [2]. Psychopathy has been linked to deficits in emotion recognition [1, 5]. It has been hypothesized that a failure to properly decode fear or sadness in others, disrupts the mechanism which normally inhibits violent behavior upon the detection of distress cues in others (violence inhibition mechanism) [6, 7]. Interestingly, studies that investigated the relationship between emotion recognition deficits, CP and CU-traits in children have produced mixed results [8–19]. It is yet unclear if emotion recognition deficits are linked to CP [8, 17, 20], CU-traits [12, 13], the interaction of CU-traits and CP [8, 9, 13], if they are not linked at all [10, 16], and if deficits are limited to specific emotions [9, 20–23] or affect all negative emotions [5, 15].

### Factors Influencing Study Results

Various factors might explain the discrepancies among study results. First, one of these factors could be differences in the experimental paradigms. Studies vary in the stimulus presentation duration (from 0.5 s to unlimited viewing time), the stimulus type (static or dynamic) as well as the response type (button press or verbal response). Longer or even unlimited viewing times could lead to speed-accuracy-trade-offs, meaning that children can compensate for deficits in emotion recognition through longer processing times. The assumption that CU-traits might influence the processing time rather than the accuracy is in line with the finding that emotion processing deficits in adults with psychopathic traits are defined by a longer processing time rather than a general failure to recognize emotions [24]. As of now, there are only two studies that measured reaction times in addition to error rates when investigating emotion recognition in children with CP and CU-traits [9, 10]. One of these studies does indicate such a trade-off [9] as participants with CP compared to typically developing (TD) participants showed a significantly lower number of errors for sadness and fear but significantly longer reaction times.

Second, different compositions of the study samples may affect the study outcome. For example, the majority of the studies with children with CP investigated the relationship between CU-traits and emotion recognition deficits in boys [10, 12, 21–23]. Only one study had a purely female sample [9] and two studies a mixed-gender study sample [8, 13]. Interestingly, these were also the only studies that reported a better fear recognition in children with high levels of CU-traits instead of a deficit. Even though these studies do not provide any direct information regarding an interaction effect of gender and CU-traits, the contrasting results indicate that gender should be considered as an influencing factor. This is further supported by the finding that females seem to be better in recognizing emotions compared to males in general [8].

Another aspect that differs among study samples, is their composition regarding CP diagnosis. Some studies only included children with CD [8] some studies included children with CD as well as ODD [9] and some children with subclinical levels of CP [21–23]. This and the heterogeneous nature of CP make it difficult to properly compare study results. Thus, instead of comparing children with and without CP, some studies opted for a dimensional approach and assessed the association between externalizing problem behavior and emotion recognition [15, 21–23]. Most of these studies included community samples and thus children with low or subclinical levels of CP [21–23]. They showed that when controlling for the level of externalizing problem behavior, CU-traits themselves predicted sadness and fear recognition deficits. However, a recent study that included children with various externalizing disorders and assessed externalizing problem behavior dimensionally [15] showed that there is an association between CU-traits and emotion recognition deficits for various emotions (sadness, fear, anger, disgust). This finding is in line with a meta-analysis, which showed that children with high levels of CU-traits show deficits in recognizing sadness, fear, and anger [5]. Thus, there is reason to assume that the association between CU-traits and emotion recognition deficits goes beyond sadness and fear but affects all negative emotions.

### The Role of Attention

It is not only unclear whether externalizing behavior or CU-traits are associated with specific or general emotion recognition deficits, but also the cause of these deficits is unknown. One cause that recent research has focused on is an irregularity in the reactivity of the amygdala [25–27]. It has been hypothesized that similar to patients with amygdala lesions [28–30], emotion recognition deficits in individuals with CU-traits and CP might be due to aberrant attention. As of now, four studies in children provide evidence that CP, as well as CU-traits, might be related to a deficit in attending towards the eyes of emotional faces [8, 12, 21, 22]. Dadds and colleagues [22] observed that high levels of CU-traits not only predicted deficits in fear recognition but also a lower number and duration of fixations on the eyes of fearful faces. In a previous study Dadds and colleagues [21] showed, that in a community sample of boys, CU-traits were negatively correlated with the accuracy in recognizing fearful facial expressions (r =  − 0.36). However, once the children were instructed to focus their attention on the eyes of the face, this association disappeared (r = 0.05) and reappeared when the children were instructed to look at the mouth (r =  − 0.24). The authors concluded that automatic misdirected attention towards the mouth instead of the eyes, in other words, a decreased eye-preference, can explain the emotion recognition deficit. To date, these results could not be replicated [8, 12]. Furthermore, the authors did not directly test the association between eye-preference and emotion recognition accuracy. For their conclusion to hold, it needs to be shown that the eye-preference level substantially mediates the relationship between CU-traits and fear recognition deficits.

To our knowledge, there are only two studies that directly investigated these associations in children with CP and CU-traits [8, 12]. Neither of these studies reported a significant association between CU-traits and fear recognition. However, Billeci and colleagues [12] found that eye-preference levels significantly mediated the association between CU-traits and sadness recognition deficits if they only analyzed children with CP, but not if they analyzed TD children. In contrast to these findings, Martin-Key et al. (2018) [8] reported that not CU-traits but CP were related to lower eye-preference levels as well as to higher emotion recognition deficits for fearful and sad facial expressions. Even though they also found that eye-preference levels were a significant predictor for the emotion recognition accuracy in their regression models, the degree to which eye-preference level explained the variance in emotion recognition accuracy was low (*f*^*2*^ < 0.05). The authors concluded that the predictive power of the level of attention towards the eyes is not sufficient to explain the emotion recognition deficits in children with CP, and thus other mechanisms must be underlying this deficit.

### The Present Study

In summary, the investigation of the relationship between CP or CU-traits with emotion recognition deficits has produced somewhat inconsistent results. Some of these inconsistencies might be due to gender differences, CP diagnosis or differences in the applied experimental paradigms. Thus, in the current study, we conducted two different emotion recognition paradigms in boys and girls with various levels of CU-traits and externalizing problem behavior. To ensure sufficient variance in the level of CU-traits and externalizing behavior, our sample consisted of TD children as well as children with a CP diagnosis. In line with previous studies, we analyzed our data dimensionally [15, 21–23]. In the first paradigm, similar to the paradigm employed by Golan and colleagues [31], children had to recognize a target emotion among three different emotional expressions. To increase participants’ motivation to answer correctly, they received auditory feedback for wrong answers and could only proceed once they chose the correct stimulus. To investigate possible speed-accuracy-trade-offs, we collected reaction times as well as error rates. The second paradigm was similar to previously conducted paradigms in which the children had to categorize the emotion of a single facial expression and were instructed to answer as fast and as correctly as possible [11–13]. During the presentation of the stimuli in this paradigm, we further collected eye-tracking data to investigate if eye-preference levels mediate the association between CU-traits and emotion recognition deficits.

In line with the meta-analytic findings [5] indicating a unique relationship between CU-traits and deficits in recognizing various emotions, we expected that higher levels of CU-traits would be associated with stronger negative emotion recognition deficits independent of the level of externalizing behavior. However, as some findings also indicated that the combination of externalizing behavior and CU-traits might influence emotion recognition [8, 9, 13], we further explored the association between the interaction of CU-traits and externalizing behavior and emotion recognition. We expected that any observed associations between CU-traits and emotion recognition deficits would be mediated by the level of attention towards the eyes.

## Methods

### Participants

A priori power analyses with the Monte Carlo Power Analysis for Indirect Effects [32] as well as g*Power 3.1.9.4 [33] were calculated. The analyses revealed that in order to test all our hypotheses with a power of at least 0.8 and medium effect sizes [5, 12], the study needed to include at least 130 participants. Thus, a total of N = 132 children aged eight to fourteen years were initially recruited for this study. Thirty participants were excluded from the final data analysis as they did not meet inclusion criteria (see below), six participants were excluded due to incomplete data, and two due to uncooperative behavior. As our dimensional approach required sufficient variance in the level of externalizing behavior and CU-traits and to better compare our results to other studies including clinical samples, we included TD children as well as children with CP in our sample. Thus, the final sample of N = 94 participants consisted of 29 children with ODD (22 male), one girl with CD and 65 typically developing children (TD) (35 male). The participants were recruited from outpatient clinics in Gießen (Germany), through advertisements in local newspapers, mainstream schools, sports clubs and via mail-shots. Inclusion criteria were: (a) no neurological or developmental disorder, (b) an IQ above 80 (c) no red-green color blindness, (d) diagnosis of CD or ODD for children with CP (according to DSM IV), (e) or no mental health problems for TD children. Of the 94 participants, 57 were male (61%). The children were all Caucasian, had a mean age of 10.42 years (*SD* = 1.74) and a mean IQ of 105.59 (*SD* = 10.75).

One parent of each child (93% mothers) completed the Observer Rating Scale for Conduct Disorder (FBB-SSV) [34] online [35] ( FBB-SSV total score: *M* = 73.32, *SD* = 4.52). If the FBB-SSV indicated CP or the child had an ODD or CD diagnosis and was referred to from a clinic, the parent completed the Kinder-DIPS [36] upon arrival at the university. CU-traits were also assessed through the online questionnaire, using the parent-version of the inventory of callous-unemotional traits (ICU) [37, 38]. ICU-total scores ranged from 17.33 to 32.95 (*M* = 22.49, *SD* = 8.94). Six children with CP took medications on the day of the experiment (three children took methylphenidates, two atypical psychotics, one hormones, see additional Table A1). 55% of the children with CP showed at least one comorbidity, ADHD (29%) being the most common one (All comorbidities of the CP-sample are presented in the additional Table A2). The study was approved by the local ethics committee. All participants and their parents gave written informed consent. Each participant received a small monetary compensation after completion of the experiment.

### Procedure

Upon arrival at the laboratory, the child first completed all computer tasks and then the IQ-test. For all computer tasks, the child was positioned in a 70 cm distance from a computer screen (DELL P2414H, 23′’, 1920 × 1080 pixel). The stimuli of all tasks were presented with *Neurobehavioral Systems* presentation software [39]. The stimuli for both tasks were taken from the *Radboud Faces Database* which was validated on a group of 276 students [40]. The models used in this database were trained according to the *Facial Action Coding System* (FACS; [41]).

### Measures

#### Semi-structured Siagnostic Interview (Kinder-DIPS)

The “Kinder-DIPS” was used to confirm CD or ODD diagnosis as well as comorbidities according to DSM IV criteria [36]. The interview has sufficient interrater reliability *(κ* = *0.48–0.88, Yule’s-Y* = *0.89–1.0)* and was conducted by psychologists in training (at least Bachelor of Science level).

#### Strength and Difficulties Questionnaire (SDQ)

The strength and difficulties questionnaire (SDQ) [42, 43] was used to assess potential mental health issues for TD children. The questionnaire consists of 25 items rated on a three-point Likert scale (0 = not at all true, 3 = completely true) and assesses internalizing as well as externalizing problem behavior. The SDQ has sufficient internal consistency (Cronbach’s alpha = 0.92). If the total problem score of the SDQ indicated abnormally high values (> 16), children were excluded from the analysis to decrease the influence of potential problem behaviors on our data.

#### Culture fair Intelligence Test (CFT-20R)

Children’s general intelligence was assessed using the short version of the CFT-20R [13, 44]. The CFT-20R shows a high internal consistency (> 0.80) and satisfying validity [44]. The short version consists of 56 items of non-verbal visual puzzles which are divided into four subtests.

#### The Inventory of Callous and Unemotional Traits (ICU)

CU-traits were assessed using the parent-version of the inventory of callous-unemotional traits (ICU) [37, 38, 45]. The ICU consists of 24 items rated on a four-point Likert scale (0 = do not agree at all, 3 = strongly agree). The ICU shows satisfactory internal consistency (Cronbach’s alpha > 0.70) and validity [46].

#### Observer Rating Scale for Conduct Disorder (FBB-SSV)

Externalizing behavior was assessed using the observer rating scale for conduct disorder (FBB-SSV) [34]. The FBB-SSV consists of 25 items rated on a four-point Likert scale (0 = not at all true, 3 = completely true). The FBB-SSV shows an internal consistency of Cronbach’s alphas = 0.89 for the entire sample, 0.90 for the ODD subscale, 0.71 for the CD subscale [34].

#### Emotion Recognition

To assess how much time children need to accurately recognize an emotion*,* we conducted an emotion recognition task [31]. In this task, the children were asked to find the target emotion among three emotional faces (angry, sad, fearful). The task consisted of three different trial blocks of 36 trials each. At the beginning of each trial block, the children were given a different target emotion (angry, sad, fearful). After the presentation of a fixation cross (two seconds), the screen showed three photographs of the same face, depicting once a sad facial expression, a fearful facial expression, and an angry facial expression. The three stimuli were presented next to each other in a randomized order. The children were asked to choose the face that depicted the target emotion, as quickly as possible. To indicate their choice the children had to press one of three buttons. Each button corresponded to the position of the stimulus on the screen (e.g. if the target emotion was depicted by the stimulus on the left, the children should press the button on the left). If they chose the correct picture, the task continued with the presentation of a fixation cross and the next trial. A short buzzing sound signaled to the children that they chose the wrong picture. In this case, they had to choose again until they made the correct decision. This was done to increase the children’s motivation to choose as fast and as correctly as possible.

All stimuli were presented in a random order within each trial block. The stimuli consisted of nine female and nine male Caucasian models. The reaction time, as well as the number of errors for each trial, were recorded.

To ensure that the children understood the task and did not generally differ in their reaction time, the emotion recognition task was preceded by a baseline task. This task was identical to the emotion recognition task but instead of choosing a target emotion among three different emotional expressions, the children were asked to choose a target color (red, yellow or blue) among three colored smiley faces. The baseline task consisted of 18 trials.

#### Emotion Categorization

To assess how well children can identify emotions in others’ facial expressions, we conducted an emotion categorization task. The task consisted of two different blocks. In the first block, the children were presented with a single emotional facial expression and they had to choose which emotion (anger, fear, sadness), the face depicted. Each trial consisted of first, the presentation of a fixation cross (four seconds), then the presentation of the emotional face (two seconds), and finally, the instruction to decide which emotion the face showed. The children indicated their choice by pressing one of three buttons, each button corresponding to one of the three emotions. The children had as much time as they needed to make their decision, however, they were instructed to try to choose as correctly and as fast as possible. The stimuli consisted of five female faces and five male faces. Thus, the first block of the task consisted of 30 trials in total (3 emotions × 2 gender × 5 faces = 30).

The second block of the emotion categorization task was identical to the first block, however, this time only the upper (eye-condition) or lower halve (mouth-condition) of the face was visible. The second block of the task consisted of 60 trials in total (3 emotions × 2 gender × 5 faces × 2 halves = 60). All stimuli were presented in random order.

#### Eye-Tracking

As a measure of the eye-preference level, children’s eye movements were tracked during the first block of the emotion categorization task. Similar to Billeci and colleagues [12], we used a remote eye-tracker from *SensoMotoric Instruments* (RED 250 Eye-Tracker). Data were collected with a binocular sampling rate of 250 Hz. Before the task, each participant performed an initial tracker calibration in which participants sequentially fixated five target points on the screen. The calibration was repeated if the accuracy did not reach the set cut-off of 0.6.

### Data Analysis

All statistical analyses were performed using SPSS version 26 [47]. For the multiple regression analyses as well as the mediation analyses the PROCESS tool was used [48].

#### Emotion Recognition Data

Five participants had to be excluded from the data analysis due to continuous inattentiveness during the task and one participant due to technical difficulties. Thus, the data analysis included 88 participants (28 CP, 60 TD). Before calculating the mean reaction time for each emotion (anger, fear, sadness), we removed individual trials in which an incorrect response was given (5.02% of all trials) as well as correct trials with reaction times reflecting impulsiveness (< 250 ms) [49] or inattentiveness (> 4000 ms) (8.45% of all correct trials). To account for individual differences in the baseline reaction time, we baseline-corrected our data by subtracting the mean reaction time during the baseline task from the mean reaction time for each emotion. The error rate for each emotion was calculated as the sum of errors over all trials. For each trial maximally one error was counted, even if the participant chose incorrectly more than once within a single trial. We employed several stepwise multiple regressions with the baseline-corrected reaction time or the error rates as the dependent variable. To investigate the unconditional influence of CU-traits and externalizing behavior (FBB-SSV total score), we included both variables as predictors in the first step of the regression. Gender and age were included as covariates, as previous findings indicated that these variables influence the processing of emotional faces [50] and as they significantly correlated with the reaction time and error rate. A previous Meta-analysis reported a positive correlation between emotion recognition abilities and IQ (r = 0.19) [51]. However, in the current study, we did not include the IQ as a covariate, as it did not significantly correlate with the reaction times or error rates. The interaction of CU-traits and externalizing behavior was added as a predictor in a second step, to investigate the moderating effect of externalizing behavior on the relationship of CU-traits and the dependent variable. As suggested by Hayes 2018 [48] we mean-centered CU-traits and externalizing behavior to render the beta coefficients of both variables interpretable.

#### Emotion Categorization Data

As the emotion categorization task was added to the experiment at a later point, twelve children of the initial sample did not perform this task and four children had to be excluded from the data analysis due to technical difficulties. Thus, the data analysis included 78 participants (24 CP, 54 TD). Similar to the analysis of the emotion recognition data we conducted several stepwise multiple regressions with the number of mistakes as dependent variable and CU-traits, externalizing behavior, age, gender and the interaction of CU-traits and externalizing behavior as predictors.

#### Eye-Tracking Data

Five children of the sample of the emotion categorization task were excluded from the data analysis due to low tracking ratios (< 50%). Thus, the data analysis included 73 participants (24 CP, 49 TD). With the help of the SMI *Begaze* software (SensoMotoric Instruments) we created two areas of interest (AOI), one around the eyes and one around the mouth of the emotional faces, in line with previous studies [8, 12, 22]. *Begaze* was also used to derive two different measures to assess eye-preference scores for each emotion: (a) fixation count: the difference between the mean number of fixations on the eye AOI and the mean number of fixations on the mouth AOI, (b) total fixation duration: the difference between the mean time spent fixating the eye AOI and the mean time spent fixating the mouth AOI. To determine whether the eye-preference level mediates the association between CU-traits and emotion recognition, we calculated two simple mediations for each emotion (model 4 of the PROCESS tool [48]). One mediation with reaction times (emotion recognition task) as the outcome variable and one mediation with error rates (emotion categorization task) as the outcome variable. In all mediations, eye preference scores served as a mediator, CU-traits as a predictor, age, gender and externalizing behavior as covariates. In cases in which our previous analyses revealed a significant interaction effect of externalizing behavior and CU-traits, we included externalizing behavior as a moderator instead of a covariate (model 5 of the PROCESS tool [48]). Indirect effects were estimated using the bootstrapping technique with 5000 bootstrap samples.

## Results

### Emotion Recognition Data

To investigate if higher levels of CU-traits are associated with stronger negative emotion recognition deficits independent of the level of externalizing behavior, we employed several stepwise multiple regressions with the baseline-corrected reaction time or the error rates as the dependent variable. As can be seen in Table 1, the higher the children’s level of CU-traits was, the longer they took to choose angry (*p* = 0.002), fearful (*p* = 0.010) or sad facial expressions (*p* = 0.020), even if the influence of age, gender and externalizing behavior and the interaction of CU-traits and externalizing behavior was kept constant. For the emotion anger, we additionally observed a negative association between the interaction of CU-traits and externalizing behavior and the reaction time (*p* = 0.029). Additional simple slope analysis revealed that CU-traits only significantly predicted the reaction time to find the angry facial expression if externalizing behavior was low, (*b* = 25.547, 95% CI [10.371, 40.723], *t* = 3.349, p = 0.001) but not if externalizing behavior was high. CU-traits did not predict the reaction time in the baseline task, thus we can exclude the possibility of principally slower reaction times for children with higher levels of CU-traits.

**Table 1:** Regression model for reaction times in the recognition task

		Fear				Sadness				Anger			
	Predictor	B	95% CI	SE	p	B	95% CI	SE	p	B	95% CI	SE	p
Step 2	age	− 1.305	[− 54.738, 52.128]	26.860	.961	− 39.534	[− 81.854, 2.786]	21.273	.067	− 21.517	[− 68.725, 25.69]	23.730	.367
	gender	− 89.835	[− 277.004, 97.333]	94.086	.342	− 12.164	[− 160.404, 136.076]	74.518	.871	12.270	[− 153.091, 177.63]	83.124	.883
	CU	15.4	[3.838, 26.961]	5.812	.010	10.895	[1.738, 20.052]	4.603	.020	16.156	[5.942, 26.371]	5.135	.002
	EXT	− 12.606	[− 35.392, 10.18]	11.454	.274	.752	[− 17.295, 18.799]	9.072	.934	− .429	[− 20.559, 19.703]	10.119	.966
	EXT*CU	− .569	[− 2.822, 1.684]	1.133	.617	− .938	[− 2.723, .846]	.897	.299	− 2.228	[− 4.218, − .237]	1.001	.029

B, unstandardized beta; SE, standard error; CU, callous-unemotional traits; EXT, externalizing behavior

The error rates in the emotion recognition task were generally very low (fear: *M* = 1.614, *SD* = 3.368; anger: *M* = 1.477, *SD* = 1.729; sadness: *M* = 2.568, *SD* = 2.896). The multiple regression analyses provided no evidence that CU-traits or the interaction with externalizing behavior can predict the error rates in recognizing angry, fearful or sad facial expressions.

### Emotion Categorization Data

Similar to the emotion recognition data, we employed several stepwise multiple regressions with the error rate as the dependent variable. The multiple regression analyses revealed that the interaction of CU-traits and externalizing behavior significantly predicted the error rates in trials in which children had to identify angry facial expressions (Table 2). An additional simple slope analysis revealed that the higher the children’s level of CU-traits was, the more mistakes they made during anger trials but only if externalizing behavior was high, (*b* = 5.513, 95% CI [0.025, 0.110], *t* = 3.185, p = 0.002), not if externalizing behavior was low. Furthermore, there was a trend (*p* = 0.052) for higher levels of CU-traits to predict a greater number of mistakes when children were asked to identify fear facial expressions. Higher levels of CU-traits also significantly predicted a generally greater number of errors (over all emotions) (*p* = 0.045).

**Table 2:** Regression model for error rates in the categorization task

	Fear				Sadness				Anger				Total			
Predictor	B	95% CI	SE	p	B	95% CI	SE	p	B	95% CI	SE	p	B	95% CI	SE	p
Whole face-condition																
Age	− .115	[− .270, .041]	.078	.145	− .026	[− .228, .176]	.101	.801	− .110	[− .282, .062]	.086	.205	− .251	[− .619, .118]	.185	.179
Gender	− .068	[− .629, .494]	.282	.810	.163	[− .568, .893]	.367	.659	.303	[− .319, .925]	.312	.335	.398	[− .935, 1.731]	.669	.554
CU	.038	[− .000, .0755]	.019	.052	.029	[− .020, .078]	.025	.246	.026	[− .016, .068]	.021	.227	.092	[.002, .182]	.045	.045
EXT	− .005	[− .068, .058]	.032	.868	− .027	[− .109, .055]	.041	.516	− .021	[− .091, .049]	.035	.550	− .053	[− .203, .097]	.075	.481
EXT*CU	.000	[− .007, .006]	.003	.920	.000	[− .008, .009]	.004	.914	.008	[.000, .015]	.004	.045	.008	[− .008, .024]	.008	.335
Eye-condition																
Age	− .200	[− .391, − .009]	.096	.040	− .269	[− .550, .012]	.141	.061	− .151	[− .347, .044]	.098	.126	− .620	[− 1.058, − .183]	.220	.006
Gender	.493	[− .198, 1.185]	.347	.159	.591	[− .426, 1.608]	.510	.250	.826	[.119, 1.533]	.355	.023	1.910	[.326, 3.495]	.795	.019
CU	.063	[.016, .110]	.023	.009	− .003	[− .072, .065]	.034	.921	.049	[.002, .097]	.024	.043	.109	[.002, .216]	.054	.046
EXT	− .038	[− .120, .040]	.039	.330	.027	[− .088, .141]	.057	.644	− .136	[− .216, − .057]	.040	.001	− .148	[− .326, .031]	.089	.103
EXT*CU	− .012	[− .020, − .004]	.004	.005	.004	[− .008, .016]	.006	.502	− .004	[− .013, .004]	.004	.337	− .012	[− .031, .007]	.009	.214
Mouth-condition																
Age	− .205	[− .461, .050]	.128	.114	− .219	[− .450, .013]	.116	.063	− .241	[− .499, .018]	.130	.068	− .664	[− 1.125, − .204]	.231	.005
Gender	.642	[− .284, 1.567]	.464	.171	− .573	[− 1.410, .2631]	.420	.176	.030	[− .906, .966]	.470	.949	.098	[− 1.567, 1.764]	.836	.907
CU	− .003	[− .065, .060]	.031	.928	.076	[.020, .133]	.028	.009	− .027	[− .091, .036]	.032	.391	.046	[− .066, .159]	.056	.416
EXT	.011	[− .093, .115]	.052	.832	.018	[− .076, .112]	.047	.702	− .044	[− .150, .061]	.053	.405	− .015	[− .202, .172]	.094	.874
EXT*CU	− .006	[− .018, .005]	.006	.244	− .002	[− .012, .008]	.005	.741	.013	[.002, .024]	.006	.024	.005	[− .015, .025]	.010	.636

B, unstandardized beta; SE, standard error; CU, callous-unemotional traits; EXT, externalizing behavior

To investigate differences between the stimuli with only the upper half of the face visible and stimuli with only the lower half visible, we calculated paired *t* tests. The tests showed that the children made significantly more mistakes in the eye-condition compared to the mouth condition for fear (eye-condition: *M* = 1.397, SD = 1.534; mouth-condition: *M* = 2.89, SD = 1.904; *p* < 0.000) and anger (eye-condition: *M* = 1.712, SD = 1.504; mouth-condition: *M* = 3.836, SD = 1.922; *p* < 0.000) but less mistakes for sadness (eye-condition: *M* = 4.74, SD = 2.007; mouth-condition: *M* = 2.986, SD = 1.897; *p* < 0.000). The results in Table 2 show that the interaction of externalizing behavior and CU-traits significantly predicted the number of mistakes in fear trials in which the children were only presented with the eyes of the emotional stimuli. As shown by the simple slope analysis, higher levels of CU-traits only predicted a greater number of mistakes if externalizing behavior was low, (*b* =  − 4.487, 95% CI [0.044, 0.188], *t* = 3.211, p = 0.002) but not if externalizing behavior was high. Furthermore, CU-traits and externalizing behavior both predicted error rates in anger trials (Table 2). Children with higher levels of CU-traits tended to make more mistakes. Children with higher levels of externalizing behavior, on the other hand, tended to make fewer mistakes if only the eyes of the anger stimuli were visible. If only the mouth of the stimuli was visible, the higher the children’s level of CU-traits the more they misidentified sad stimuli.

### Eye-Tracking Data

To investigate whether attention towards the eyes mediates the relationship between CU-traits and emotion recognition (reaction times) or categorization (error rates) a mediation model for each emotion was calculated. As the interaction of externalizing behavior and CU-traits significantly predicted the recognition and categorization of anger stimuli, externalizing behavior was included as a moderator in the mediation analyses of anger stimuli. For all other analyses, externalizing behavior was included as a covariate. None of the bootstrap confidence intervals for the indirect effects were entirely above zero. Thus, the mediation analyses provide no evidence that a preference to look at the eyes mediated the association between CU-traits and the recognition or categorization of emotional faces. The two eye-preference measures did not significantly predict the reaction times or error rates. As can be seen in Table 3, the two eye-preference measures were only significantly associated with gender, indicating that girls showed higher eye-preference levels than boys.

**Table 3:** Mediation model for eye-preference level

	Fear				Sadness				Anger			
Predictor	B	95% CI	SE	p	B	95% CI	SE	p	B	95% CI	SE	p
Fixation time												
Age	1.187	[− 1.784, 4.158]	1.489	.428	1.012	[− 2.215, 4.239]	1.617	.534	.780	[− 2.370, 3.929]	1.578	.623
Gender	− 12.196	[− 23.044, − 1.3480]	5.436	.028	− 11.886	[− 23.669, − .102]	5.905	.048	− 10.671	[− 22.171, .830]	5.763	.068
CU	.046	[− .607, .700]	.328	.888	− .079	[− .789, .630]	.356	.824	− .019	[− .711, .674]	.347	.958
EXT	− .275	[− 1.496, .9466]	.612	.655	− .274	[− 1.600, 1.052]	.665	.681	− .280	[− 1.575, 1.015]	.649	.668
Fixation count												
Age	.027	[− .107, .162]	.067	.686	.029	[− .111, .169]	.070	.676	− .012	[− .147, .123]	.068	.861
Gender	− .740	[− 1.231, − .249]	.246	.004	− .684	[− 1.195, − .173]	.256	.009	− .653	[− 1.146, − .160]	.247	.010
CU	.000	[− .029, .030]	.015	.981	.000	[− .031, .031]	.015	.983	.004	[− .026, .034]	.015	.801
EXT	− .025	[− .080, .030]	.028	.371	− .032	[− .089, .026]	.029	.274	− .025	[− .080, .031]	.028	.379

B, unstandardized beta; SE, standard error; CU, callous − unemotional traits; EXT, externalizing behavior

## Discussion

With the current study, we tested whether externalizing problem behavior and CU-traits are associated with emotion recognition deficits of negative emotional faces, using two different experimental paradigms. We further investigated whether found associations between emotion recognition deficits and CU-traits would be mediated by the level of attention participants pay to the eyes of an emotional stimulus. One aim of the current study was to take different factors into account which might have influenced previous results. Thus, we used two different experimental paradigms. One paradigm, which included additional measures to increase participants’ motivation to answer correctly and allowed for an analysis of potential trade-off effects between processing time and error rate and another paradigm without such measures. Furthermore, to account for differences in group composition, gender was added as a covariate in the analysis and we included children with and without CP but analyzed the data dimensionally to reduce the influence of the heterogeneity of CP and to account for subclinical levels of externalizing behavior.

We observed different results in the two different emotion recognition paradigms. As expected, CU-traits were associated with increased reaction times to fearful, sad and angry stimuli in the emotion recognition task. Thus, in line with previous studies [5, 15] CU-traits seem to be associated with emotion processing deficits for various emotions and not just sadness and fear. However, neither in the emotion recognition task nor the categorization task did CU-traits significantly predict the error rates. This could be due to various reasons.

First, the number of mistakes may have decreased in exchange for an increase in reaction time. This assumption would be in line with the findings indicating that the emotion recognition deficit in adults with psychopathic traits lies in a longer processing time rather than a general failure to recognize emotions [24]. Trade-offs between processing time and error rates could also be one of the reasons for discrepancies among studies with differing stimulus presentation times. Dadds and colleagues [15] for example presented the stimuli for only 500 ms and, in keeping with our findings, reported a relationship between CU-traits and a recognition deficit for all negative emotions. The participants in the study by Woodworth and Waschbusch [13] on the other hand, had an unlimited viewing time, opening the possibility for a trade-off between processing time and error rates. Such a trade-off could, for example, explain their finding of better fear recognition in children with high levels of CU-traits. As suggested by Vitale and colleagues [24], the problem of a slower emotion processing, in contrast to a general problem in recognizing emotions in others, might be susceptible to treatment. Thus, emotion recognition training targeting the processing speed of children with high levels of CU-traits might help them to encompass their insufficiencies.

Second, the tasks might have been too easy to detect a relationship between CU-traits and error rates. Even though previous studies conducted similar tasks with children of the same age [11–13] and reported deficits in emotion recognition, the children of our study made very few mistakes in general. Nonetheless, they made considerably fewer mistakes in the recognition task compared to the categorization task (relative to trial number 36 vs. 10). Furthermore, there was a trend (p = 0.052) for an association between CU-traits and more mistakes in recognizing fear in the emotion categorization task. Thus, the emotion recognition task may have been easier than the emotion categorization task. In general, our results indicate that reaction times might be a more sensitive method to detect differences in the emotion recognition ability than error rates.

Even though there was no significant relationship between error rates and CU-traits in either of the emotion recognition tasks, we observed a significant relationship between the interaction of CU-traits and externalizing problems and the error rate for angry stimuli in the second paradigm, indicating that CU-traits were associated with a higher error rate if the level of externalizing problems was high as well. This result is rather surprising as previous studies have only reported a relationship between having CP and a reduced ability to recognize angry facial expressions but no interaction effects [8, 14, 20]. Interestingly, there was also an interaction effect of CU-traits and externalizing problems in the first paradigm. However, this interaction indicated that CU-traits were associated with longer reaction times to angry stimuli if the level of externalizing behavior was low. Thus, this could indicate that children with high levels of CU-traits and high levels of externalizing behavior have a general problem with recognizing angry stimuli and children with high levels of CU-traits and low levels of externalizing behavior have deficits in their processing speed. Due to the differences among the paradigms, this assumption needs to be considered with caution. As previously mentioned, the paradigms might have differed regarding their level of difficulty. Also, the emotion recognition task may have been less influenced by motivational aspects. In contrast to the categorization task, wrong choices in the recognition task were followed by a buzzing sound and the task only continued once a correct choice was made. Thus, the children might have been more motivated to make the right choice in the emotion recognition task compared to the categorization task.

In the second part of our study, we investigated whether the found associations between CU-traits and emotion recognition deficits are mediated by the participant’s level of attention on the eyes. Similar to Han and colleagues [26] and in line with the assumption that the eyes are the most salient feature for the recognition of fear [52], we observed a better recognition for fear and anger over the whole sample, when only the eyes were presented compared to trials in which only the mouth was visible. Given the finding that the eyes compared to the mouth receive the most attention in sad stimuli [53] our finding of a higher number of mistakes in the eye-compared to the mouth-condition for sad faces is surprising. In accordance with the finding that the relation between CU-traits and fear recognition deficits seems to decrease, once children are instructed to look at the eyes of fearful faces [21], we expected a similar result when we presented the children with only the eyes of the fearful stimuli. However, putting the eyes in the focus of attention did not decrease the relationship between CU-traits and a fear recognition deficit. Consistent with this finding, our eye-tracking results do not provide any evidence suggesting that the eye-preference level mediates the emotion recognition deficits in children with high levels of CU-traits. Comparable to Martin-Key and colleagues [8] we did not observe a significant association between eye-preference and CU-traits for either of the three emotions. Thus, the longer processing time in children with high levels of CU-traits does not seem to be associated with a lack of attention to the eyes of emotional faces. In line with Martin-Key et al. [8] and previous studies in healthy adults [54], we also observed significant gender effects with a higher eye-preference level for girls compared to boys. Interestingly, Billeci and colleagues [12] who only investigated boys, observed a significant mediation of eye-preference on CU-traits and the recognition of sad facial expressions in their CP-group. Thus, it is possible that eye-preference levels only contribute to the emotion recognition deficits in boys with high levels of CU-traits and CP but not in girls. As of yet, all the studies that investigated the relationship of CU-traits, emotion recognition and attention to the eyes used a cross-sectional study design [8, 12, 21]. Even though, Dadds and colleagues [21] established the causal relationship of attention to the eyes and emotion recognition deficits by showing that emotion recognition deficits can be reduced through instructing the children to look at the eyes of an emotional facial expression, the causal relationship between CU-traits and attention to the eyes remains unclear. As causal inferences in mediation analysis can only be withdrawn if the temporal ordering of the variables in a mediation model is correct [48, 55], longitudinal studies would be needed to establish whether CU-traits are a result of or a cause of aberrant attention to the eyes.

## Strengths and Limitations

The present study is the first study to investigate emotion recognition deficits using two different paradigms and one of few studies to directly investigate the influence of eye-preference levels on the relationship between CU-traits and emotion recognition deficits in a mixed study sample. Thus, our study not only replicates and extends previous results on the relationship between CU-traits, externalizing problem behavior, emotion recognition, and attention but further delivers valuable information about different aspects that influence the results of emotion recognition tasks, which might explain discrepancies among previous study results.

Including the measurement of reaction times in our emotion recognition task allowed us to ensure appropriate task performance and reduce the influence of impulsive or inattentive behavior. We believe that the assessment of reaction times, the buzzing sound signaling a wrong choice, as well as the trial repetition until the correct choice was made contributed to an increased willingness to properly participate in the task and thus led to a reduction of the influence of motivational aspects.

Aside from these strengths, several limitations need to be mentioned. Six participants with CP took medication on the day of the experiment, however, we did not have sufficient power to compare performance among children with and without medication. Thus, we do not know in how far medication might have influenced children’s performance. Additionally, we cannot tell whether our attempts to increase the children’s motivation to follow task instructions in the emotion recognition task was successful, as we did not have an objective measure of the level of motivation. However, in light of the fact that children with externalizing problems have problems with motivation [9, 56, 57] and as indifference to one's performance is a symptom characterizing CU-traits, future studies should consider the influence the motivation to follow task instructions has on the study results.

To properly assess eye-movements, we had to use a fixed stimulus presentation time in the emotion categorization task, which is why we could not assess reaction times in this paradigm. The inclusion of the measurement of reaction times in this paradigm would have been important to support our findings of the emotion recognition task indicating that CU-traits are associated with longer processing times rather than deficits in recognizing emotions. Before each trial of the categorization task, we presented a fixation cross to refocus the attention of the participants on the center of the screen. This fixation cross was positioned slightly closer to the mouth AOI than the eye AOI, hence, we had to exclude the first fixation from our analysis and were not able to investigate potential differences in the first initial shift. According to a study in patients with amygdala lesion [30], emotion recognition deficits may be related to a lack of the first automatic shift towards the eyes instead of differences in the total fixation duration on the eyes. However, others who investigated CU-traits and initial eye-preference did not observe meaningful relations [8]. Furthermore, due to the use of static facial expressions, our study has a rather low ecological validity. Future studies would benefit from including dynamic facial expressions in the investigation of a potential failure to attend to the eyes.

As indicated by a priori power analyses, the mediation analysis would have required at least 130 participants. As the final analysis only included 73 participants, we cannot exclude the possibility that we were unable to observe a significant mediation effect of attention on the association between CU-traits and emotion recognition due to insufficient power. However, post hoc power analyses showed that with a medium effect size settled at f^2^ = 0.15 [5] and p = 0.05 we achieved a power > 0.8 for all our other analyses.

## Summary

The results of the current study provide evidence that under consideration of the influence of gender, age and the level of externalizing problem behavior, high levels of CU-traits in children are associated with slower but not less accurate performance in recognizing sad, angry and fearful facial expressions. There were no indications that the longer processing time might be due to a lack of attention towards the eyes of emotional facial expressions. However, we found that girls compared to boys tended to spend more time looking at the eyes of emotional faces. Thus, future studies should investigate the possibility that eye-preference levels only contribute to the emotion recognition deficits in boys with high levels of CU-traits but not in girls.

## Electronic supplementary material

Below is the link to the electronic supplementary material.

Supplementary file1 (DOCX 58 kb)

## Data Availability

The datasets used and/or analyzed during the current study are available from the corresponding author on reasonable request.
