# Supplementary material for: Emotion Processing in Children with Conduct Problems and Callous-Unemotional Traits: An Investigation of Speed, Accuracy, and Attention
Source: Child Psychiatry Hum Dev. 2020 Mar 13;51(5):721–33. doi: 10.1007/s10578-020-00976-9 (PMC7518997; doi:10.1007/s10578-020-00976-9)
Supplement: Supplementary file 1 — Supplementary file1 (DOCX 58 kb) [file 10578_2020_976_MOESM1_ESM.docx]

**Emotion processing in children with conduct problems and callous-unemotional traits: An investigation of speed, accuracy, and attention**

Daniela Hartmann^1^, Christina Schwenck^1^

^1^ Justus-Liebig-University of Giessen, Department of Special Needs Educational and Clinical Child and Adolescent Psychology

Corresponding author:

Daniela Hartmann

Otto-Behaghel-Straße 10 C

D-35394 Gießen

Tel. : +49 641 99 26176

Fax : +49 641 99 26019

<mailto:daniela.hartmann@psychol.uni-giessen.de>

**Supporting information**

Table A1: *Correlations of all variables of the emotion recognition analysis.*

|  | Age | | Sex | | CU | | EXT | |
| --- | --- | --- | --- | --- | --- | --- | --- | --- |
|  | r | p | r | p | r | p | r | p |
| Afraid (RT) | -.112 | .150 | -.044 | .341 | .249 | .010 | -.068 | .265 |
| Sad (RT) | -.285 | **.004** | .027 | .402 | .294 | **.003** | .025 | .408 |
| Angry (RT) | -.206 | **.027** | .085 | .215 | .304 | **.002** | -.018 | .434 |
| Afraid (error rate) | .015 | .446 | .100 | .177 | .163 | **.065** | .195 | **.034** |
| Sad  (error rate) | -.089 | .205 | .291 | **.003** | .010 | .463 | .075 | .243 |
| Angry (error rate) | .029 | .393 | .215 | **.022** | -.016 | .440 | -.114 | .145 |

r, pearson correlation coefficient; RT, reaction time; CU, callous-unemotional traits; EXT, externalizing behavior

Table A2: *Correlations of all variables of the emotion categorization analysis.*

|  |  | Age | | Sex | | CU | | EXT | |
| --- | --- | --- | --- | --- | --- | --- | --- | --- | --- |
|  |  | r | p | r | p | r | p | r | p |
| Whole face condition | Afraid  (error rate) | -.245 | **.015** | .007 | .477 | .302 | **.004** | .067 | .281 |
|  | Sad  (error rate) | -.061 | .297 | .084 | .232 | .177 | .061 | -.014 | .452 |
|  | Angry  (error rate) | -.170 | .069 | .135 | .120 | .345 | **.001** | .096 | .201 |
| Eye condition | Afraid  (error rate) | -.276 | **.007** | .168 | .070 | .223 | **.025** | -.104 | .183 |
|  | Sad  (error rate) | -.207 | **.035** | .108 | .172 | .141 | .109 | .107 | .176 |
|  | Angry  (error rate) | -.176 | .061 | .241 | **.017** | .187 | **.050** | -.301 | **.004** |
| Mouth condition | Afraid  (error rate) | -.160 | .080 | .124 | .140 | -.003 | .491 | -.015 | .450 |
|  | Sad  (error rate) | -.324 | **.002** | -.099 | .194 | .368 | **.000** | .134 | .121 |
|  | Angry  (error rate) | -.194 | **.044** | -.053 | .321 | .060 | .300 | -.022 | .425 |

r, pearson correlation coefficient; RT, reaction time; CU, callous-unemotional traits; EXT, externalizing behavior

|  | Afraid (FD) | | Sad (FD) | | Angry (FD) | | Afraid (FC) | | Sad (FC) | | Angry (FC) | | |
| --- | --- | --- | --- | --- | --- | --- | --- | --- | --- | --- | --- | --- | --- |
|  | r | p | r | p | r | p | r | p | r | p | r | p |  |
| age | .049 | .339 | .044 | .356 | .027 | .412 | -.007 | .476 | .003 | .489 | -.077 | .260 |  |
| sex | -.264 | **.012** | -.252 | **.016** | -.231 | **.025** | -.365 | **.001** | -.331 | .**002** | -.331 | **.002** |  |
| CU | -.096 | .209 | -.132 | .133 | -.102 | .196 | -.142 | .116 | -.143 | .114 | -.086 | .234 |  |
| EXT | -.093 | .217 | -.102 | .195 | -.093 | .218 | -.158 | .090 | -.180 | .064 | -.137 | .125 |  |
| Afraid (RT) | .118 | .160 | .157 | .093 | .089 | .226 | .233 | **.024** | .267 | .**011** | .145 | .111 |  |
| Angry (RT) | .112 | .172 | .109 | .178 | .089 | .228 | .200 | **.045** | .169 | .077 | .118 | .159 |  |
| Sad (RT) | .117 | .162 | .137 | .124 | .100 | .200 | .219 | **.031** | .160 | .088 | .075 | .265 |  |

Table A3: *Correlations of all variables of the eye-tracking analysis.*

r, pearson correlation coefficient;FD, mean fixation duration; FC, mean fixation count; RT, reaction time; CU, callous-unemotional traits; EXT, externalizing behavior

A4: *Examplary questions of the Observer Rating Scale for Conduct Disorder (FBB-SSV).*

Externalizing behavior was assessed using the observer rating scale for conduct disorder (FBB-SSV) [1]. The FBB-SSV consists of 25 items rated on a four-point Likert scale (0 = not at all true, 3 = completely true).

| Item |  | Not at all true | Somewhat true | Mostely true | Completely true |
| --- | --- | --- | --- | --- | --- |
| 3 | Often gets into fights with adults. | 0 | 1 | 2 | 3 |
| 5 | Often provokes others intentionally. | 0 | 1 | 2 | 3 |
| 13 | Tortures animals. | 0 | 1 | 2 | 3 |
| 15 | Steals money or valuables. | 0 | 1 | 2 | 3 |

References

1. Döpfner M, Görtz-Dorten A, Lehmkuhl G et al. (2008) Diagnostik-System für Psychische Störungen Nach ICD-10 Und DSM-IV fürKinder Und Jugendliche-II (DISYPS-II)
